# Immune escape mechanisms of severe fever with thrombocytopenia syndrome virus

**DOI:** 10.3389/fimmu.2022.937684

**Published:** 2022-07-28

**Authors:** Tong Wang, Ling Xu, Bin Zhu, Junzhong Wang, Xin Zheng

**Affiliations:** ^1^ Department of Infectious Diseases, Union Hospital, Tongji Medical College, Huazhong University of Science and Technology, Wuhan, China; ^2^ Joint International Laboratory of Infection and Immunity, Huazhong University of Science and Technology, Wuhan, China

**Keywords:** SFTSV, immune escape, innate immune response, adaptive immune response, SFTS

## Abstract

Severe fever with thrombocytopenia syndrome (SFTS), which is caused by SFTS virus (SFTSV), poses a serious threat to global public health, with high fatalities and an increasing prevalence. As effective therapies and prevention strategies are limited, there is an urgent need to elucidate the pathogenesis of SFTS. SFTSV has evolved several mechanisms to escape from host immunity. In this review, we summarize the mechanisms through which SFTSV escapes host immune responses, including the inhibition of innate immunity and evasion of adaptive immunity. Understanding the pathogenesis of SFTS will aid in the development of new strategies for the treatment of this disease.

## Introduction

SFTS is an emerging infectious disease that was first identified in rural areas of China in 2009 and has since been reported in Japan, South Korea, Vietnam, Taiwan, and many other countries and regions (1–3). The epidemiology shows that SFTS is more common in older people, mostly farmers, and mainly transmitted through the bite of infected ticks. The pathogen of this disease, SFTSV, also known as Dabie bandavirus, can infect a variety of domestic and wild animals, in addition to humans. The clinical manifestations of SFTS include sustained high fever with thrombocytopenia, leukopenia, gastrointestinal symptoms, and multiple-organ failure, with a mortality rate of up to 30% (4–7).

SFTSV, a member of the genus *Bandavirus*, belongs to the family *Phenuiviridae* of the order *Bunyavirales* (8). The surface envelope of this spherical virion is mainly composed of two transmembrane glycoproteins and lipid bilayers with spinous processes. SFTSV is a single-stranded, negative-sense, enveloped RNA virus whose genome comprises three segments: large (L), medium (M), and small (S) (8, 9). The L segment encodes a viral RNA-dependent RNA polymerase, which is necessary for viral replication, whereas the M segment encodes a glycoprotein precursor (Gn/Gc), which is cleaved into the Gn and Gc viral proteins by a host cell protease. Gn/Gc can promote virus invasion into host cells by binding to the cell surface C-type lectin DC-SIGN or the host cell receptor non-muscle myosin heavy chain IIA and mediate virus–cell membrane fusion with high antigenicity (2, 10–12). The S segment belongs to the ambisense RNA family and has two reading frames in opposite directions. The 3′ end reverse sequence encodes a nucleoprotein (NP), which mediates viral ribonucleoprotein complex formation, whereas the 5′ end sequence encodes a nonstructural (NSs) protein, which can inhibit the host innate antiviral immune response (2, 13, 14).

The host immune response determines the clinical manifestation of SFTSV infection (15). In immunocompetent patients, a robust immune response can be elicited to eliminate virus infection. However, in older or immunocompromised patients, SFTSV escapes from host immune surveillance or cannot be eliminated by the weak immune defense (15–20). An effective vaccine or antiviral agents that could be potential approaches to prevent and treat SFTSV infection are not available (15, 21). Thus, exploring the mechanisms through which SFTSV evades host immune responses can be useful for the development of vaccines or therapeutics against this viral infection. In this review, we summarize and discuss how STFSV escape from host immunity.

## Innate immune evasion by SFTSV

The innate immune response acts as the first line of defense against SFTSV infection. However, the virus can evade the innate immune response through affecting the number and function of innate immune cells, inhibiting interferon (IFN) production and function, interfering with nuclear factor-κB (NF-κB) signaling, and regulating autophagy.

### Attenuated function of mononuclear cells and DCs by SFTSV infection

Monocytes are important members of innate immune cells. They recognize pathogenic microorganisms through TLRs, induce the expression of cytokines, chemokines, and synergistic stimulation molecules, and differentiate into macrophages or myeloid DCs (mDCs) to initiate innate or adaptive immune responses. Monocytes also participate in regulating the function of plasma cell-like DCs (pDCs), and TNF-α secreted by pDCs plays an important role in the antiviral immune response (22, 23). It has been found that monocytes may be one of the main target cells of SFTSV infection (23–25). Decreased numbers and dysfunction of monocytes in patients with acute SFTS are related to the severity of the disease (23).At the early stage of fatal SFTSV infection, Song et al. (26) found that monocytes underwent severe apoptosis and necrosis. In addition, compared to patients in the recovery stage and healthy controls, the responsiveness of SFTSV-infected monocytes was significantly weakened against LPS stimulation, indicating that these cells are immunologically incompetent.

In addition, Cong et al. has revealed that SFTSV can infect macrophages directly and exist in splenic macrophages much longer than in other organs in a rodent model (27). The effect of SFTSV on macrophage varies at different stages of infection. Zhang et al. (28) confirmed that in the early stage of infection, SFTSV induced a monocyte immune response and stimulated macrophages to differentiate into the M1 phenotype by activating STAT1, resulting in the production of proinflammatory cytokines (such as TNF-α,IL-1β,IL-6) and tissue destructive. At a later stage, SFTSV infection could upregulate the expression of IL-10 and activates STAT3. STAT3 binds to the promoter of the pre-miR-146b gene to promote the production of miR-146b, which can inhibit the differentiation of M1 macrophages and drive macrophages to differentiate into the M2 phenotype by targeting STAT1, thereby promoting virus shedding and causing virus transmission. Furthermore, it has been demonstrated that viral NSs protein is a component of SFTSV to mediate the increased miR-146 expression.

DCs are the most important antigen-presenting cells. They serve as the bridge between innate and adaptive immune responses and are an essential component of the host’s defense system. DCs in the human peripheral blood are mainly divided into mDCs and pDCs (29). In patients with lethal SFTSV infection, the mDC apoptosis rate is significantly increased after SFTSV infection (26). In addition, the expression of IL-4 and GM-CSF is necessary for the differentiation and maturation of DCs, whereas these cytokines are significantly downregulated in monocytes and lymphocytes, especially in severe and lethal cases (30, 31). The marked elevated apoptosis rate of the mDC by SFTSV infection, along with mDC maturation disorders resulting from massive monocyte apoptosis and IL-4 and GM-CSF deficiency in deceased patients with SFTS, finally leading to a significantly decreased population of effective DCs. Meanwhile, the insufficiency of mDCs impedes the differentiation of naïve T cells into T follicular helper cells (Tfhs) (26, 32). Zhang et al. (33) confirmed that the decrease in the number of mDCs positively correlated with the severity of the disease. In addition, both pDCs and mDCs express an important PRR, TLR3. When activated by double-stranded RNA during viral replication, they can quickly produce large amounts of type I IFNs (34). However,the TLR3 expression by mDCs in deceased group exhibited gradually downregulated during the acute phase (35). Previous studies have shown that DC maturation relays on the TLR3 signaling pathway. It has been observed that in surviving patients with SFTS, high levels of mDCs are directly proportional to TLR3 expression in CD14^+^HLA-DR^+^ monocytes (35, 36). This suggests that virus-mediated downregulation of TLR3 expression in CD14^+^HLA-DR^+^ monocytes may lead to the suppression of mDC differentiation, which eventually leads to an apparent decrease in IFN production and the weakening of the cellular immune response (23, 35). Furthermore, it has been found that effective differentiation of mDCs, but not pDCs, plays a key role in the antiviral immune response in patients with SFTS (35). These studies suggest that SFTSV infection could abate the function of monocytes and DCs in immune responses and induce the differentiation of macrophages into the M2 phenotype, ultimately facilitating virus persistence and disease progression.

### Reduction of NK cell subsets number by SFTSV

NK cells are effector cells of the innate immune system that can directly or indirectly kill virus-infected cells by releasing perforin and granzyme, as well as proinflammatory cytokines and chemokines in the early stage of infection. These cells also participate in antibody-dependent cell-mediated cytotoxicity. In addition, NK cells play an important immunomodulatory role in the adaptive immune response (37–39). Individuals with impairment of NK cell numbers and functions are more susceptible to virus infections, including cytomegalovirus (CMV), herpes simplex virus (HSV), ect (37).

Based on the expression intensity of the surface markers CD16 and CD56, NK cells in human peripheral blood can be divided into the following five subgroups: CD56^bright^CD16^−^, CD56^bright^CD16^+^, CD56^dim^CD16^−^, CD56^dim^CD16^+^, and CD56^−^CD16^+^. CD56^dim^CD16^+^ NK cells are the dominant population and account for at least 90% of all peripheral blood NK cells (40). In the early stage of SFTSV infection, the frequencies of CD56^dim^CD16^+^ NK cells were greatly decreased and negatively correlated with disease severity (41). In addition, the expression of Ki-67 and granzyme B levels were enhanced, and NKG2A expression was decreased in CD56^dim^CD16^+^ NK cells after acute infection. Furthermore, compared with that in the recovery phase in patients with severe SFTS, the effector function of CD56^dim^CD16^+^ NK cells were enhanced in the acute phase (41). Thus, despite the reduction of NK cells, the function and activation of CD56^dim^CD16^+^ NK cells were enhanced. Liu et al. (42) observed that in the phase 3 of SFTSV infection (≥11 days after the onset of symptoms), the counts of CD3^−^CD16^+^CD56^+^NK cells decrease significantly compared to the healthy controls. NK cells are crucial at resisting viral infection, which depletion in patients with SFTS hinders viral clearance and the host immune response, thereby weakening the antiviral effect and aiding host immune response evasion by SFTSV.

### Inhibition of the IFN signaling pathway by SFTSV

IFNs play critical roles in antiviral immune response and can modulate the innate and adaptive immune responses. IFNs are mainly divided into three types: I, II, and III. Of these, type I IFN(IFN-I) are important components of the innate immune response. They mainly include IFN-α and IFN-β, which are secreted by infected cells, such as macrophages, dendritic cells (DCs), and epithelial cells (43). When host cells are infected with SFTSV, their pattern recognition receptors (PRRs) firstly recognize pathogen-associated molecular patterns and subsequently induce the production of IFN-I and IFN-inducible proteins. Toll-like receptors (TLRs) and retinoic acid-inducible gene I (RIG-I)-like receptors are the major PRRs. RIG-I-like receptors, including RIG-I and melanoma differentiation-associated gene 5(MDA5), recruit mitochondrial antiviral-signaling protein, which transmits the signal of TANK-binding kinase 1 to activate the NF-κB and IFN regulatory factor 3 pathways, and synergistically promote the expression of IFN-I genes (44). Among TLRs, TLR3, TLR7, and TLR8 have been identified as viral RNA sensors in endosomes (45). Activated TLRs recruit specific linker molecules, TIR domain-containing adaptor-inducing IFN-β and myeloid differentiation factor 88, to trigger the production of inflammatory factors and IFN-I (46). IFN-I is secreted extracellularly and subsequently binds to membrane receptors (IFNAR1 and IFNAR2) on the surface of effector cells, activating the JAK/STAT signaling pathway and ultimately promoting the expression of IFN-stimulated genes (43).

Several animal model studies found that immunocompetent adult mice or hamsters did not show obvious infection symptoms after SFTSV infection. However, SFTSV-infected mice that were deficient in the α/β IFN receptor (IFNAR^−/−^) and Syrian hamsters that were deficient in the gene encoding signal transducer and activator of transcription 2 (STAT2^−/−^) exhibited high serum viral loads and a hematological status which is similar to that of human infection, and the infection resulted in death. Therefore, IFN-I signaling is critical for preventing lethal SFTSV infection (6, 47–50).

SFTSV can interfere the production of IFN-I through multiple mechanisms.Studies have found that the SFTSV NSs protein could specifically trap TRIM25 into inclusion bodies (IBs) and impede TRIM25-mediated Lys-63 ubiquitination and activation of RIG-I, leading to the inhibition of the host antiviral response (51, 52). Khalil J et al. found that the C-terminus of the SFTSV NSs protein could specifically bind to TANK-binding kinase 1 to form cytoplasmic IBs, which is also known as viroplasm-like structures. The IBs formed by SFTSV can not only be used as a viral replication factory but also capture important protein molecules of the IFN signaling pathway (such as TANK-binding kinase 1, IKKϵ, and the IFN regulatory factor 3 complex), thereby hindering the generation of IFN-I and promoting virus replication (53). Another experiment found that the SFTSV NSs protein could also hijack mitochondrial antiviral-signaling protein into IBs, thereby hindering the IFN signaling pathway and inhibiting the activation of NF-κB signaling. In addition, SFTSV NP could inhibit the IFN-β response by interfering with the IFN regulatory factor 3 and NF-κB signaling pathways, thereby enhancing viral replication (24, 54). Furthermore, analysis of immune-related genes in SFTSV-infected peripheral blood mononuclear cells showed that the expression of TLR3 in deceased patients was downregulated nearly 10 times compared with that in healthy controls in the acute phase, and the expression of IFN-α1/β1 mRNAs in the TLR3 signaling pathway was downregulated as the severity of SFTS increased (35).

During the IFN signal transduction phase, the SFTSV NSs protein can inhibit IFN signaling and the expression of ISG by sequestering STAT2 and STAT1 into IBs and impairing the phosphorylation and nuclear translocation of STAT2 heterodimers (55–57). Another study has demonstrated that SFTSV NSs protein could suppress both type I and III IFN signaling by inhibiting STAT1 phosphorylation and activation (58). Ning et al. (59) and Gough et al. (60) showed that IFN-γ (type II IFN) had anti-SFTSV efficacy *in vivo* and *in vitro*. The SFTSV NSs protein could also hijack STAT1 into viral IBs and decrease its expression, thereby inhibiting the type II IFN response. Thus, mounting evidence indicates that the SFTSV NSs protein is a key factor involved in virus evasion from the host innate immune response. Further studies are needed to clarify the role of the NP in this process.

### The effect of NF-κB signaling on SFTSV replication

As an important transcription factor, NF-κB has a variety of biological functions during viral infection, such as proinflammatory, antiviral, and apoptotic responses. NF-κB activation is mediated *via* the canonical and noncanonical pathways. According to previous studies, the virus can evade or activate inflammation through multiple mechanisms involving NF-κB signaling. Qu et al. (24) confirmed that the NF-κB signaling pathway might be temporarily activated during the early stages of SFTSV infection in THP-1 cells, which promoted viral replication. However, pretreating THP-1 cells with an NF-κB inhibitor before SFTSV infection reduced the viral load (24). Subsequently, the role of NF-κB was gradually attenuated, and further experiments showed that both the SFTSV NSs protein and NP could inhibit the NF-κB signaling pathway. Studies in HeLa cells also confirmed the suppressive effect of the SFTSV NSs protein on NF-κB signaling (61, 62). By contrast, the study by Sun et al. (62) showed that the SFTSV NSs protein activated the NF-κB promoter activity in NSs-overexpressing HepG2 cells, resulting in the promotion of NF-κB-dependent proinflammatory responses in SFTSV-infected cells. However, pretreating HepG2 cells with the NF-κB inhibitor Bay11-7082 significantly reduced not only the expression of proinflammatory cytokines but also the number of S gene copies in cells (62). Using an established minigenome system based on the M segment of SFTSV, Mendoza et al. (63) confirmed that the NF-κB inhibitor SC75741 reduced the synthesis of viral proteins and replication of SFTSV *in vitro*. Furthermore, another study found that SFTSV triggered the activation of NF-κB signaling *via* the TLR8–myeloid differentiation factor 88 axis during virus entry (64). Taken together, these results indicate that the activation of the NF-κB signaling pathway by the SFTSV NSs protein is cell- or tissue-specific and restrained NF-κB signaling can reduce the viral load in infected cells and tissues. At present, the mechanisms of NF-κB signaling promoting SFTSV replication is not well understood and should be further studied.

### Promotion of SFTSV replication by regulating autophagy

The human innate immune system can defend against viral infection through a variety of mechanisms, one of which is the elimination of intracellular pathogens by host cells through autophagy. In addition, heterogeneous autophagy can directly activate PRRs and trigger their signal transduction pathways, thereby promoting NK T-cell activation, cytokine secretion, and phagocytosis. Autophagy also plays an important role in maintaining immune homeostasis (65). However, during evolution, many viruses have developed the ability to evade antiviral immune responses *via* regulating autophagy (66, 67). During SFTSV infection, autophagy can not only be induced and utilized as a platform for virus replication and assembly but also for the release of the progeny viruses through autophagy-related vesicles to enhance SFTSV infection (68, 69). SFTSV may affect autophagy through the following mechanisms. SFTSV-induced autophagy depends on the NP, which can directly inhibit the interaction between BECN1 and BCL2 and induce complete Becn1-dependent autophagy flux (68). SFTSV assembly relies on phagocytic vesicles formed by endoplasmic reticulum–Golgi intermediates. The assembled progeny SFTSV particles can survive in autophagy-related vesicles (such as autophagosomes and autophagolysosomes) and are released directly outside the cell through the exocytosis of autophagic vesicles (68, 70). In another study, transfection of HeLa and HepG2 cells with the VR1012-GFP-LC3 recombinant plasmid confirmed that autophagy was a promoting factor of SFTSV replication (71). Sun et al. (72) found that the SFTSV NSs protein could colocalize with the autophagy-related proteins LC3, p62, and Lamp2b under SFTSV-infected status but failed to colocalize alone with them, indicating that conventional autophagosomes are not equivalent to SFTSV NSs protein-induced IBs. In addition, the LC3-II protein was accumulated in SFTSV-infected cells, and treatment with a lysosomal protease inhibitor did not affect the results, which showed that SFTSV replication might be promoted *via* inhibition of autophagic degradation (69, 72). Furthermore, viruses can restrain the cell cycle by interacting with host factors. The SFTSV NSs protein can inhibit the formation and nucleation of the cyclin B1–CDK1 complex by interacting with CDK1 and inducing cell cycle arrest in the G2/M phase to promote virus replication and release (71). To date, studies have confirmed that SFTSV infection can induce autophagy and utilize autophagy to promote self-replication, the underlying interaction between SFTSV and autophagy is not completely clear, and the effect of autophagy on various antiviral signaling pathways (such as the IFN signaling pathway) should be studied in the future.

## SFTSV evasion of the adaptive immune response

Host adaptive immune responses against viruses involve T and B cells, which specifically recognize and eliminate viral pathogens. T cells are lymphocytes involved in cellular immunity. When stimulated by antigens, T cells are transformed into sensitized lymphocytes and elicit specific immune responses. B cells are the main cells involved in humoral immunity and are transformed into immunoglobulin-producing plasma cells under antigenic stimulation. Antibodies produced by plasma cells can not only kill infected target cells *via* antibody-dependent cell-mediated cytotoxicity but also directly neutralize viruses (73). SFTSV can achieve immune escape by affecting T- and B-cell immune responses through different mechanisms.

### Involvement of T-cell depletion and subset disorder in SFTSV infection

T lymphocytes are the main immune cells that mediate cellular immune responses. According to previous studies (18, 74, 75), the numbers of T lymphocytes, especially CD4^+^ T lymphocytes, which limit the initiation and maintenance of humoral and cytotoxic T-cell immunity, are significantly reduced in surviving and deceased patients with SFTS during the acute phase. Compared with those in the healthy control group, the numbers and proportions of CD4^+^ T cells in surviving and deceased patients were found to be significantly reduced, and the reduction in the deceased patients was even worse than that in the surviving patients (74). Although the CD8^+^ T-cell numbers were also reduced, the reduction was not significantly different between the surviving and deceased patients (74). Therefore, the reduction in the number of CD4^+^ T cells is a more meaningful marker for evaluating disease severity and prognosis. Consistently, the numbers of T helper (Th) 1, Th2, and regulatory T (Treg) cells in the deceased patients were significantly lower than those in the surviving patients, whereas the number of Th17 cells in the deceased patients was higher than that in the surviving patients in the early stage of SFTS (74). Th17 cells can inhibit the ability of effector T cells to kill target cells, thereby hindering the host’s antiviral response (76). The decrease in the numbers of Th1, Th2, and Treg cells and the relative increase in that of Th17 cells seriously affect their regulatory roles in cellular and humoral immunity and lead to a disorder of immune function and excessive release of inflammatory cytokines (74). Moreover, it has been shown that PD-1 expression on dysfunctional CD4^+^ and CD8^+^ T cells is significantly increased during the acute stage of SFTSV infection, which may weaken the antiviral T-cell immunity (75). Meanwhile, in the early stage of acute SFTSV infection, T cells not only actively proliferate but the effector function of CD4^+^ T cells and cytotoxic function of CD8^+^ T cells are enhanced (75).

A previous study has found that the expression levels of apoptosis markers (annexin V and CD95) on CD4^+^ and CD8^+^ T cells in infected individuals during the early stage of SFTSV infection were significantly higher than those in the healthy population (75); therefore, apoptosis may also be one of the reasons for the decrease in the numbers of CD4^+^ and CD8^+^ T cells. Fas/FasL interactions may also play an important role in T-cell apoptosis.

Apoptosis is a strictly regulated physiological process that is closely related to innate immunity. When SFTSV replicates in host cells, host-induced apoptosis of the infected cells is an effective antiviral mechanism that can efficiently inhibit the spread of the virus. Bortezomib (PS-341) is an effective and selective FDA-approved proteasome inhibitor, which has been shown to suppress SFTSV replication by interfering with the apoptosis pathways in 293T cells (52). Similar results were obtained when SFTSV-infected cells were treated with the apoptosis-inducing agent STS (52). Proteomic analysis revealed that some antiapoptotic proteins, such as SOD2, BCL3, CD74, and FAM129B, were upregulated during SFTSV infection, indicating that SFTSV infection exerts an antiapoptotic effect (64). In addition, Hou et al. (77) found that Th17 cells could upregulate antiapoptotic molecules to promote continued replication of the virus in SFTSV-infected cells. However, apoptosis has a dual effect on the host antiviral response. Apoptotic death can be induced not only in infected cells to inhibit virus replication but also in a large number of immune cells, which leads to the weakening of the antiviral effect. Hence, SFTSV can escape immune responses by inhibiting T cell immune response and regulating the apoptosis of SFTSV-infected cells.

### Inhibition of antibody secretion and the maturation of B cells by SFTSV infection

The humoral immune response after viral infection not only neutralizes the virus by producing antibodies but also prevents viral reinfection (78). The B-cell immune response is regulated by antigen-presenting cells and Tfhs (32). However, SFTSV infection can impair antigen-presenting function of B cells and mDCs, as well as impair Tfhs, resulting in a significantly weakened humoral immune response (24, 26, 35). Using single-cell RNA sequencing analysis of SFTS patient PBMCs, Angela et al. (79) showed that the B-cell population obviously expanded, which was associated with disease severity (26, 79, 80). Moreover, study has suggested that B-cell lineage populations are targets for SFTSV, and B cells, specifically plasma cells, are potential virus reservoirs in the blood of patients with fatal SFSTV infections (79). A postmortem analysis of lymph nodes has further confirmed that the majority of SFTSV-infected cells are B cells, specifically plasmablasts (PBs) (80). It is speculated that PBs may first be infected by SFTSV in lymph nodes and then differentiate into plasma cells, which are then circulated in the blood. By contrast, SFTSV may independently infect plasma cells in lymph nodes and blood (79). Furthermore, compared with those in uninfected B cells within the fatal group, the IFN and sirtuin signaling pathways are significantly downregulated and the NRF2-mediated oxidative stress response is upregulated in SFTSV-infected B cells (79). This reduction in IFN signaling is probably due to the IFN-antagonistic function of the viral NSs protein, which further promotes virus infection and replication. However, IFN and sirtuin signaling were elevated in the entire B-cell population of the fatal group (79). Another study has revealed that PBs in fatal cases proliferated to a greater extent than those in survived ones (26). However, the pronounced expansion of PBs contrasts to the decrease in the production of specific antibodies. Song et al. (26) found that deceased patients exhibited a complete absence of both serum IgM and IgG specific to the SFTSV NP, as well as the absence of Gn-specific IgG. Meanwhile, surviving patients developed IgM antibodies to the SFTSV NP in the acute stage of infection, and NP-specific IgG antibodies appeared 2 to 3 weeks after symptom onset (26). Accumulated evidences has indicated that the proliferation of PBs is dysfunctional and the effective humoral immune response is impaired in fatal SFTS because of the failed antibody class-switch response (26, 80). Therefore, SFTSV infection may inhibit antibody secretion and the maturation of B cells, thus suppressing effective humoral responses for the virus to escape from the host immune system.

## Conclusion

**Figure 1:**
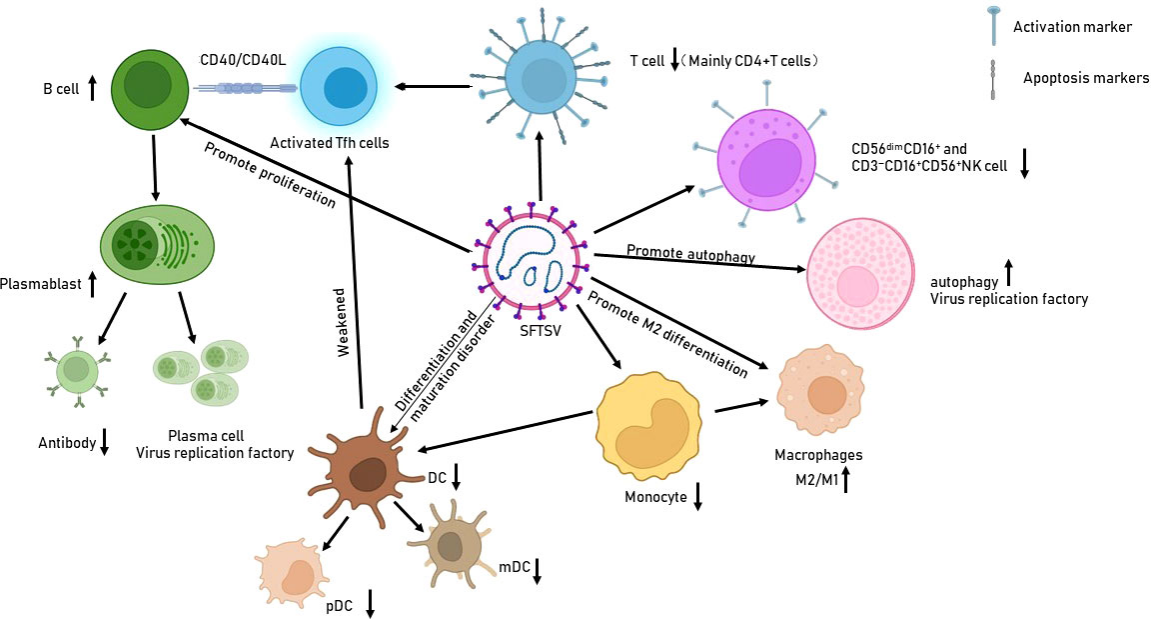
Effects of SFTSV infection on immune cells. SFTSV infection impairs the production and function of various immune cells, such as monocytes, macrophages, and DCs. It also promotes B-cell proliferation and differentiation into PBs/plasma cells, which are deemed potential virus reservoirs.

**Figure 2:**
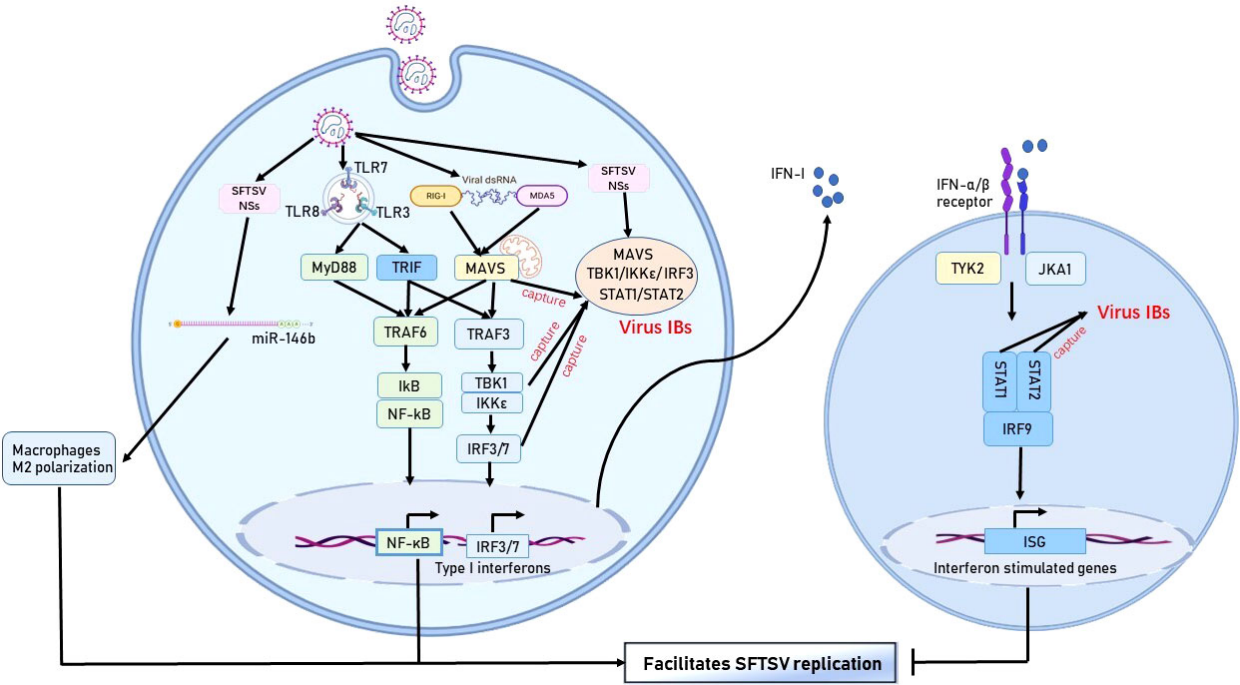
Immune escape mechanism of SFTSV in monocytes. The SFTSV NSs protein hijacks various protein molecules of the IFN pathway by forming IBs, thereby hindering IFN production. In addition, SFTSV activates the NF-κB pathway and promotes macrophage differentiation into the M2 phenotype to facilitate viral replication.

Increasing evidence indicates that SFTSV can escape from host immune responses *via* multiple strategies: interfering the number and function of innate and adaptive immune cells (**Figures 1**, **2**), inhibiting of the IFN signaling pathway (**Figure 2**), regulating the NF-κB signaling (**Figure 2**) and autophagy, ect. Modulating dysfunctional immune cells should be the potential strategy to rescue host immune clearance against this virus. Understanding the mechanisms of immune evasion by SFTSV is critical for the development of a vaccine and immunotherapy.

## Author contributions

XZ and JW designed and planned the work and revised the manuscript. TW and LX performed the literature search and interpretation and manuscript drafting. BZ revised the manuscript. All authors contributed to the article and approved the submitted version.

## Funding

This study was supported by the Key Biosafety Science and Technology Program of the Hubei Jiangxia Laboratory (JXBS001); the National Science and Technology Major Project of China (92169121); the Applied Basic and Frontier Technology Research Project of Wuhan (2020020601012233); the Fundamental Research Funds for the Central Universities (2020kfyXGYJ016); and the National Natural Science Foundation of China (81501748); Innovation Team Project of Health Commission of Hubei Province (WJ2019C003).

## Conflict of interest

The authors declare that the research was conducted in the absence of any commercial or financial relationships that could be construed as a potential conflict of interest.

## Publisher’s note

All claims expressed in this article are solely those of the authors and do not necessarily represent those of their affiliated organizations, or those of the publisher, the editors and the reviewers. Any product that may be evaluated in this article, or claim that may be made by its manufacturer, is not guaranteed or endorsed by the publisher.
